# Sarcoidosis: Progression to the chronic stage and pathogenic based treatment (narrative review)

**DOI:** 10.3389/fmed.2022.963435

**Published:** 2022-09-06

**Authors:** Anna Malkova, Yulia Zinchenko, Anna Starshinova, Dmitriy Kudlay, Igor Kudryavtsev, Anzhela Glushkova, Piotr Yablonskiy, Yehuda Shoenfeld

**Affiliations:** ^1^Laboratory of the Mosaic of Autoimmunity, St. Petersburg State University, Saint Petersburg, Russia; ^2^Phthisiopulmonology Department, St. Petersburg Research Institute of Phthisiopulmonology, Saint Petersburg, Russia; ^3^Almazov National Medical Research Centre, Saint Petersburg, Russia; ^4^Department of Pharmacology, I.M. Sechenov First Moscow State Medical University, Moscow, Russia; ^5^Laboratory of Personalized Medicine and Molecular Immunology, NRC Institute of Immunology FMBA of Russia, Moscow, Russia; ^6^Department of Immunology, Institution of Experimental Medicine, Saint Petersburg, Russia; ^7^V.M. Bekhterev National Research Medical Center for Psychiatry and Neurology, Saint Petersburg, Russia; ^8^Sackler Faculty of Medicine, Ariel University, Ariel, Israel; ^9^Zabludowicz Center for Autoimmune Diseases, Sheba Medical Center, Tel-Hashomer, Israel

**Keywords:** sarcoidosis, pathogenesis, fibrosis, autoimmunity, immune therapy, adjuvant, autoimmune diseases

## Abstract

Many factors confirm the autoimmune nature of sarcoidosis and help in determining the strategy of patient management and treatment initiation. However, the causes and the mechanisms of disease progression that result in fibrosis and insufficiency of the affected organ remain unclear. This narrative review aims to analyse the mechanisms and biomarkers of sarcoidosis progression, as well as the pathogenetic basis of sarcoidosis therapy. The following characteristics of progressive chronic sarcoidosis were revealed: the disease develops in patients with a genetic predisposition (SNP in genes GREM1, CARD15, TGF-β3, HLA-DQB1^*^06:02, HLA-DRB1^*^07/14/15), which contributes either the decreased ability of antigen elimination or autoimmune inflammation. Various prognostic biomarkers of disease progression (decreased levels of neopterin, elastase, sIL-2R, chitotriosidase, glycoprotein Krebs von den Lungen, Th17 cell count, reduced quantity of TNF-α in peripheral blood or bronchoalveolar lavage fluid) have been described and can potentially be used to determine the group of patients who will benefit from the use of corticosteroids/cytostatic drugs/biologics.

## Introduction

According to the current concept, sarcoidosis occurs as a result of an exposure to various exogenous or endogenous antigens in subjects with a genetic predisposition to autoimmune disorders and is associated with the development of non-necrotizing granulomas in different organs (1).

The search for etiologic factors of sarcoidosis has revealed various infectious agents (from bacteria, viruses to fungi) as well as non-organic factors (e.g., silicone, silicates etc.) that could be associated with its development (2, 3). Long-term exposure to these factors is believed to cause a chronic over-stimulation of the immune response and chronic inflammation. Since no specific etiologic factor has been established, the pathogenesis of the disease remains poorly understood; furthermore, it appears impossible to create a disease model, develop a unified approach to therapy, and conduct non-clinical studies for the assessment of treatment efficacy (4).

Based on the pathologic findings, sarcoidosis is currently believed to be a granulomatous disease that can be either acute, subacute or chronic and is most commonly (in >90% of cases) associated with the involvement of the lungs and mediastinal lymph nodes as well as other organs and tissues and the development of non-necrotizing granulomas (1, 5, 6). It is known that a severe progressive course of sarcoidosis can lead to a failure of the affected organ, which requires enhanced therapy or even transplantation of healthy tissues (7). For this reason, it is important to understand the disease pathogenesis to inform patient management.

The aim of this review is to analyse the mechanisms and biomarkers of sarcoidosis progression, as well as the pathogenetic basis of sarcoidosis therapy.

### Search strategy and selection criteria

The data for the narrative review were collected from the studies published in international databases MEDLINE, Current Contents, PubMed, Elsevier between 2001 and 2021 using the search words of “sarcoidosis, progression, pathogenesis, genetic markers, laboratory markers, treatment.” Inclusion criteria were original research with observation of patients with progressive sarcoidosis and comparison group (group without progression or the studied group before the progression), review articles and research articles on pathogenesis of sarcoidosis.

## Granuloma formation

**Figure 1:**
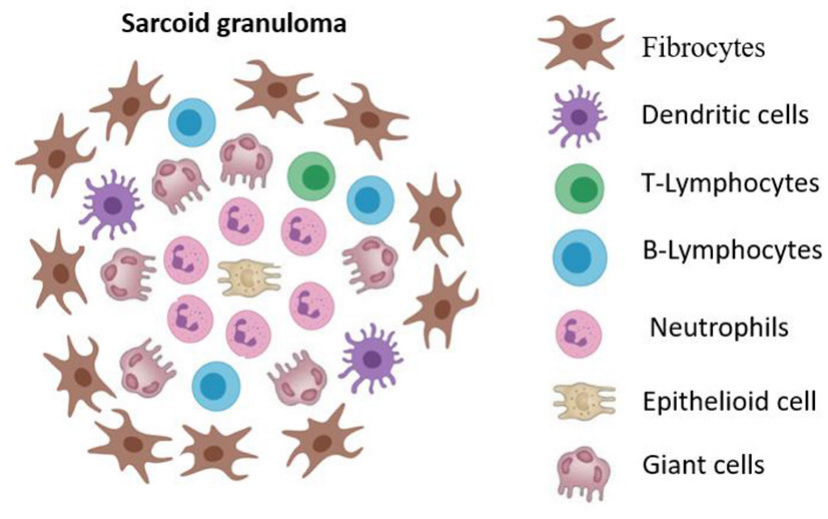
The cellular structure of sarcoid granuloma. Non-caseating epithelioid granuloma represented by lymphocytes, epithelioid and giant cells (asteroid and conchoidal bodies). The central part of a granuloma is composed of macrophages, modified macrophages, epithelioid cells and giant cells, with CD4+ T-lymphocytes between them. The peripheral part of granuloma is predominantly occupied with CD8+ T lymphocytes, fibroblasts, macrophages and fibrocytes.

The key feature of the pathogenesis of sarcoidosis is the formation of granulomas in the lungs, mediastinal lymph nodes, skin and other organs. In patients who are genetically predisposed to this disease, a contact of antigen-presenting cells (macrophages, dendritic cells, epithelial cells) with an unknown foreign antigen results in the dysregulated immune response that is manifested as granulomatous inflammation (8). The main characteristic of sarcoidosis is the formation of non-caseating epithelioid granulomas in various organs, represented by lymphocytes, epithelioid and giant cells (asteroid and conchoidal bodies) (Figure 1). Unlike infectious-mediated granulomas in sarcoidosis, necrotic masses are not formed and ACE hyperproduction occurs (9, 10). The central part of a granuloma is composed of macrophages, modified macrophages, epithelioid cells and giant cells, with CD4+ T-lymphocytes between them (11). The peripheral part of granuloma is predominantly occupied with CD8+ T lymphocytes, fibroblasts, macrophages and fibrocytes. B lymphocytes are very rare in a granuloma usually (12). Central fibrinoid might be found (10).

Macrophages, dendritic cells and epithelial cells are the first cells to react with the antigens due to the presence of Toll-like receptors. Long-term exposure of Toll-like receptors to foreign antigens results in activation and epithelioid differentiation of macrophages, which then start producing pro-inflammatory cytokines (TNF-a, IL-1). Having bound to the antigen, dendritic cells migrate to lymph nodes, where they present the antigen to T-lymphocytes. Further activation of immune cells involves various chemokines such as CCL5 (for Th1) and CCL2 (for Th2). Much attention has been recently paid to the role of Th17 lymphocytes in the pathogenesis of sarcoidosis. These cells are a CD4+ lymphocyte subset that express IL-17A and have pro-inflammatory or anti-inflammatory properties as a result of the local inflammatory environment. Granuloma macrophages in patients with sarcoidosis were shown to express CCR20 thus attracting Th17 cells, while IL-23 expression causes a significant increase in IL-17A concentrations (13, 14). Since there is growing evidence of high plasticity of Th-cells (including Th17), the hypothesis of specific differentiation of T helper cells that are responsible for the initiation of granuloma formation and/or the development of chronic granulomas should be withdrawn.

The predominant phenotype among macrophages in sarcoidosis granulomas is M2, which have anti-inflammatory function and contribute to the disease progression. Moreover, *in vitro* and *in vivo* models have also demonstrated the presence of M2 at the initial stages of granuloma formation. There is growing evidence of the role of mTOR signaling pathway in the granuloma formation demonstrated in studies of molecular mechanisms of macrophage dysfunction. The activation of mTORC1 in murine macrophages was shown to result in the disease progression and formation of granulomas just as in humans with progressive sarcoidosis (14, 15). Metabolic adaptation to the environment and inflammatory conditions profoundly affects the regulation of autophagy, leading to the impaired antigen clearance and contributing to the persistence/progression of the granulomas.

More recent data also support the concept that sarcoidosis is a disorder associated not only with Th1 immune response but also with a potential dysfunction of regulatory immune cells and immune system impairment related to the incapability to eliminate the antigen, which is evidenced by the absence of necrotic tissue (16). The functional cell properties such as *T*-cell depletion may be as relevant cause of the progression of sarcoidosis as the profile of cytokine secretion.

The interaction of macrophages and T-cells and their relationship with the microenvironment (rather than individual mediators) should be the main objectives of disease profiling determining future personalized therapeutic approaches (17).

## Genetic predisposing factors

The development of a chronic disease is associated with individual characteristics of the immune system indicating the existence of some genetic predisposing factors. Several candidate genes have been described in literature:

### *GREM1* gene

The human gene *GREM1* encodes gremlin, a highly conserved glycoprotein, a member of the BMP antagonist family (Members of the TGF-β superfamily), including TGF-βs and bone morphogenetic proteins (BMPs). Heron et al. (18) found that sarcoidosis patients carrying the CC genotype of GREM1 had 6.4 times increased risk of fibrosis development.

### CARD15 gene

The caspase recruitment domain (CARD) 15 gene (nucleotide oligomerisation domain (NOD)2) encodes an intracellular protein CARD15 of the NOD family. These proteins are involved in innate immunity through recognition of muramyl dipeptide, a component of bacterial cell wall peptidoglycan (19). It was shown that patients carrying the 2104T (702W) polymorphism of CARD15 gene were more likely to have radiographic stage IV disease at 4-yr follow-up. All patients possessing both CARD15 2104T and the C-C chemokine receptor (CCR)5 gene HHC haplotype had stage IV disease at presentation (20).

### TGF-β genes

In the study of Kruit et al. the genes encoding isoforms of the transforming growth factor β were analyzed in patients with different stages of sarcoidosis. The frequency of TGF-beta3 4875 A, 17369 C alleles, the TGF-beta2 59941 G allele were significantly higher in sarcoidosis patients with pulmonary fibrosis developing than in patients with acute or /self-limiting and chronic sarcoidosis (*p* = 0.04; corrected *p* = 0.2; OR, 2.9) (21).

### HLA-genes

The literature search showed that HLA-DQB1^*^06:02, HLA-DRB1^*^07/14/15 genotypes were associated with chronic sarcoidosis (22).

The predisposing genes are summarized in the Table 1.

**Table 1:** Genetic predisposing factors of sarcoidosis progression.

**The gene**	**Function**	**The role in sarcoidosis**
*GREM1*	Encodes gremlin (BMP antagonist family)	CC genotype-increased risk of fibrosis
CARD15	Encodes CARD15 (NOD family)	2104T (702W) polymorphism - higher in patients with radiographic stage IV disease
TGF-β	Encodes TGF-β	The TGF-beta3 4875 A, 17369 C, the TGF-beta2 59941 G - higher in fibrotic patients
HLA	Encodes human leukocytes antigens	HLA-DQB1*06.02, HLA-DRB1*07/14/15 genotypes – higher in patients with chronic sarcoidosis

In the research conducted by Lockstone et al. it was revealed that genes involved in leukocyte activation and differentiation, and cytokine production were overexpressed in patients with a progressive, fibrotic (P-F) pulmonary disease compared with the self-limiting group. Among these processes most significant changes were revealed in intracellular signaling (NF-kB and JAK-STAT cascades) and cell life (apoptosis, cell cycle, cell proliferation, and homeostasis) (23).

## The pathogenesis of sarcoidosis progression

In some cases when it is impossible to eliminate the pathogen or chronic autoimmune inflammation develops (6, 24–26), collagen overproduction in the area of granulomas is induced, which eventually causes fibrosis and substitution of lung tissue with connective tissue. Fibrotic changes start at the periphery of granulomas and extend centrally (27).

The causes of fibrosis are unknown. However, some characteristics have been described in patients with chronic sarcoidosis.

### Alterations of granuloma cell differentiation

Biopsy specimens obtained in patients with a progressive disorder demonstrated Treg with BTNL2 and ANXA11 mutations whose gene products have anti-inflammatory and immune regulatory properties. It is possible that Treg downregulate the effective inflammation and contribute to the development of chronic sarcoidosis (28, 29).

The role of Th2 in the progression of sarcoidosis is being actively studied. It has been suggested that a transition from a Th1 to a Th2 cytokine signature may occur in chronic sarcoidosis, perhaps as a response to persistent inflammation (30). The patients also showed an increase in IL-13 mRNA expression, which is one of the key cytokines of Th2 cells in peripheral blood (31). Animal studies (32) and tests of tissue samples obtained from sarcoidosis patients (33) demonstrated that the overproduction of Th2 cytokines was associated with an activation and differentiation of tissue macrophages with predominant formation of M2 cells. These cells contribute to the development and maintenance of the sites of chronic tissue inflammation, granuloma and fibrosis formation (34). The cytokines produced by Th2 cells - IL-4 and IL-13 - activate the STAT6 pathway in macrophages, with subsequent induction of numerous downstream mediators (34).

M2 macrophage polarization, as reflected by an increased CD163 expression, is associated with an enhanced antigen presentation within a select subset of regulatory T cells, leading to lower concentrations of IFN-γ and higher concentrations of IL-10 upon stimulation. Moreover, IL-13 has anti-inflammatory properties, including suppression of TNF-α release.

### Cytokine production

IL-13 can stimulate macrophages to release TGF-β (35). In patients with a new-onset sarcoidosis, differences in lung lavage granulocyte counts and TGF-β release have been detected between those who eventually undergo clinical remission and those who develop a chronic disease course (36).

IL-13 and CCL2 secreted by Th2 were implicated in the pathogenesis of lung fibrosis models, including in idiopathic pulmonary fibrosis (37, 38) (Table 2). CCL2 promotes fibroblast survival, and also has been demonstrated to be elevated in sarcoid lung samples (39, 40).

**Table 2:** The cytokines and its role in sarcoidosis progression.

**The cytokine**	**The origin**	**The role in progression**
IL-13	Th2	Stimulate macrophages to release TGF-β
CCL2		Promotes fibroblast survival
TGF-β	Macrophages	Contribute to collagen production by fibroblasts
Arginase		
CCL18	Antigen presenting cells	Contribute to collagen production by Fibroblasts supports a M2 polarization of macrophages

CCL18 has been directly implicated in fibrosis (41). CCL18 production is associated with fibroblast stimulation and augmented collagen production (42). In sarcoidosis, increased CCL18 was associated with sarcoidosis-associated pulmonary fibrosis but not with other phenotypes of pulmonary disease. CCL18 supports a M2 polarization of macrophages in sarcoidosis-associated pulmonary fibrosis and suggests a pathogenic role of these macrophages in the fibrotic response.

M2 polarized macrophages preferentially express Arg1, which results is an increased arginase. Arginase metabolizes arginine to ornithine, a precursor for the collagen substrate proline (43). Th2 cytokine signature is thought to promote M2 polarization and was shown to correlate with the expression of Arg1 and with the development of fibrosis (44).

Cytokines released during the granuloma formation and macrophage transformation directly activate JAK-STAT pathway, which supports chronic inflammation (45). High production of IFN-γ induces a STAT1 in granuloma macrophages (46) while IL-6 – STAT3 in surrounding lymphocytes, which was confirmed by immunohistochemistry analysis of tissues of patients with cutaneous sarcoidosis (47).

**Figure 2:**
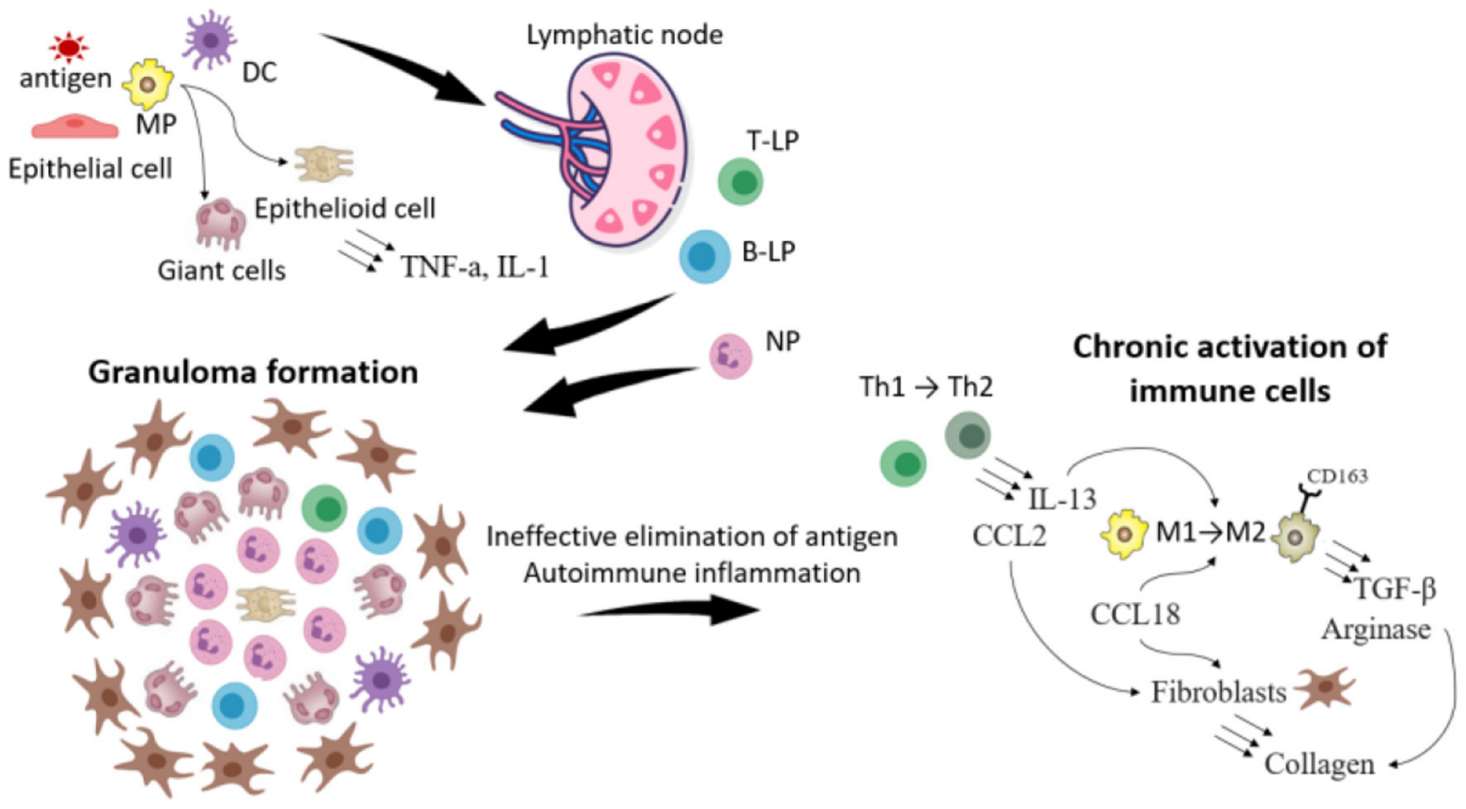
The scheme illustrating the progression of sarcoidosis. The foreign antigen is captured by macrophages (MP) and dendritic cells (DC). DC migrate to lymphoid nodes, where they present the antigen to T- and B-lymphocytes (T-, B-L). Meanwhile in the location of the inflammation MP because of the disability to eliminate the antigen transform to giant and epithelioid cells. T, B-L and neutrophils (NP) migrate in the location of the inflammation and the granuloma is formatted. Because of the impairment of the immune response (weak functions of leukocytes or in opposite the development of autoimmune reactions) immune cells are chronically activated, which leads to the change of their phenotype. Thus T-helper cells (Th1) of type 1 transform to type 2, which secrete IL-13 and CCL2. IL-13, CCL18 contributes to the transformation of MP type 1 to type 2 (expressing CD163), which produce TGF- β and arginase. All these processes stimulate synthesis of collagen by fibroblasts, which leads to the sarcoidosis progression and fibrosis.

The current understanding of sarcoidosis progression is shown in Figure 2.

## Animal models

Animal models of sarcoidosis are difficult to induce, the previous attempts to evaluate the causes of the disease progression and the etiology-based treatment were unsuccessful (48). Animal studies have mainly demonstrated the development of pulmonary granulomas without fibrosis following exposure to various antigens (*M. Tuberculosis* peptides*, P. Acne*, carbon nanoparticles). In various studies, mice have developed multiple areas of granulomatosis (lungs, skin, liver, stomach, ganglia), which may indicate the features of the immune response in healthy mice, allowing for more effective elimination of pathogens. The granulomas observed in the experiment were shown to exist only if there was a continuous exposure to the adjuvant. We believe that sarcoidosis patients are chronically exposed by one or several triggers that contribute development and persistence of granulomas (48) or autoimmune inflammation (6).

Some models have shown similar tissue abnormalities as in patients with a chronic sarcoidosis. The authors of a study in a murine model with tuberous sclerosis complex 2 (TSC2) knockout gene described the development of granulomas showing epithelioid-like macrophages, M2 macrophage polarization, and mTORC1 pathway activation (15). TSC2 downregulates mTOR complex 1 (mTORC1) by inhibiting inflammatory immune responses in innate immune cells, such as monocytes, macrophages and dendritic cells. Genetic manipulation of the mTOR pathway in mice alters macrophage polarization and the production of inflammatory and immunomodulatory cytokines (49). Analysis of biopsy material showed an activated mTORC1 pathway significantly enriched (FDR < 0.001) and a significantly decreased mRNA expression of TSC1 in patients with the progressive relative to the self-limiting form of the disease (15).

## The predictors of the progression of sarcoidosis

Some pathogenesis-related changes in the pulmonary tissue are accompanied by alterations in peripheral blood and bronchoalveolar lavage fluid in patients with progressive sarcoidosis. Accordingly, the observed correlation between some parameters and the development of granuloma inflammation in the tissues has revealed markers for sarcoidosis progression, namely increased levels of neopterin molecule synthesized from macrophages, sIL-2R marker of T-cell activation, chitotriosidase secreted by active macrophages, neutrophil-derived collagenase and elastase (50), and a low level of TNF-α synthesized from alveolar macrophages and T-cells (51).

Increased number of Th17 in peripheral blood (52) and higher bronchoalveolar (BAL) neutrophil elastase concentrations (36) were more characteristic for patients with progressive sarcoidosis. In addition, a number of studies have shown higher levels of neutrophils in BAL in patients with more advanced stages of the disease (53). Sarcoid granulomas are known to demonstrate a high glucose uptake (54), therefore in these loci increased glycolysis is observed (15). An increase in the concentration of Krebs von den Lungen-6 (KL-6) glycoprotein was observed and was often associated with fibrosis (51).

Some clinical characteristics such as extrapulmonary manifestations (especially cardiac sarcoidosis or neurosarcoidosis), composite physiologic index (CPI) - the index of lung function variables, which correlates with the degree of interstitial disease and CT measurements - the degree of fibrosis, enlargement of the pulmonary artery and the measures of diffuse lung carbon monoxide (DLCO) percentages were shown as markers of severity of pulmonary sarcoidosis (36, 48, 55, 56).

The use of Scadding radiographic stages can also predict the prognosis, the stage I disease was associated with the spontaneous resolution in 60 to 90% patients, while with an increase of stages the percentage of resolution possibility decreased (up to 0% in stage IV fibrotic disease) (57).

The whole list of possible laboratory and clinical markers predicting the progression of sarcoidosis is presented in Table 3.

**Table 3:** The predictor markers of the progression of sarcoidosis.

**Laboratory markers (blood/BAL)**	**Clinical markers**
•↑ neopterin •↑ sIL-2R •↑ chitotriosidase •↑ neutrophil-derived collagenase and elastase •↓ TNF-α •↑ Krebs von den Lungen-6 (KL-6) glycoprotein •↑ number of Th17	•Extrapulmonary manifestations (especially cardiac sarcoidosis or neurosarcoidosis) •Composite physiologic index (CPI) •Enlargement of the pulmonary artery •The measures of diffuse lung carbon monoxide (DLCO) •Increase of scadding radiographic stages

## Pathogenesis-based rationale for sarcoidosis therapy

In some patients, sarcoidosis may be associated with a failure in the affected organ, which requires treatment. However, a substantial proportion of patients are likely to subsequently develop a chronic or progressive sarcoidosis, an estimated percentage of 10% to 20% of patients will have sequelae, and a fatal outcome is observed in 6–7% of cases (58). The aim of the sarcoidosis therapy is the prevention of progression, fibrosis and the organ failure. Predicting the course of sarcoidosis during the initial assessment is a difficult task in clinical practice (59). A more detailed understanding of the pathogenesis of disease progression can help to formulate the criteria for prescribing therapy and improve understanding the mechanism of its effects.

Regulation of the immune response, as well as prevention of fibrosis due to suppression of the overactive macrophages, T-cells and cytokine (TNF-a, IL-1, IFN-γ and IL−6) production can be the main approaches of the sarcoidosis treatment (60, 61).

### Drugs directed against autoimmune component

Current first-line therapy for sarcoidosis include corticosteroids (prednisolone, methylprednisolone) (62). The mechanism of action of this drug group is based on their rapid and potent anti-inflammatory activity as a result of suppression of cytokines (including *TNF-*α*, INF-*γ) that contribute to the development of granulomas (63, 64). The most suitable dosage and treatment regimen (from 15 mg in cases of lung involvement to 20–40 mg or more in patients with generalized disease and life-threatening conditions) have not been determined (62).

Recent studies have showed evidence for a role for humoral immunity in the sarcoidosis development indicating the possible use of anti-B-cell therapy. Therapy with anti-CD20 monoclonal antibody rituximab was associated with clinical improvement in sarcoidosis patients, which is most likely due to the role of “naive” and/or B- memory cells in the development of sarcoidosis (65–67). The exact role of rituximab as a third- or fourth-line therapy agent remains unclear, and further studies of B-cell immune response are required.

The prescribing factor for these therapy could be the finding of autoantibodies on BAL or blood serum, such as autoantibodies to mutated citrullinated vimentin (anti-MCV) (68, 69), and imbalance in B-cell population with the decrease in memory cells and increase in naïve and regulatory B-cells, which is common for autoimmune diseases (62, 63).

### Drugs directed against immune cell differentiation

Second-line therapy includes cytotoxic agents (methotrexate, azathioprine, leflunomide, mycophenolate, as well as their combinations, mainly a combination of methotrexate and leflunomide). The main objective of the use of this group of drugs is to overcome the adverse effects of corticosteroids, taper their dose up to complete discontinuation (steroid-sparing effect). Methotrexate is the most commonly used second-line therapy agent. Methotrexate is a folic acid antagonist, which suppresses the production of TNF-α through adenosine A2A receptors, simultaneously inducing IL-4 and IL-13 that are up-regulators of M2 polarization. MTX can also polarize M0 to M2 *via* IL4-independent pathways. Thus, the desirable anti-inflammatory effects of MTX are leveled by an increased risk of fibrosis (70, 71).

Leflunomide, a dihydroorotase inhibitor, can be used alone or in combination with methotrexate, including in patients with generalized, progressive disease or with incomplete effect from other therapy. Leflunomide affects the inflammatory process by inhibiting Th17 cells and activating Tregs (72). Azathioprine affects RNA and DNA synthesis by inhibiting purine metabolism, suppresses lymphocyte proliferation, decreases the production of circulating T- and B-cells, and increases apoptosis in circulating lymphocytes. However, the exact mechanism of azathioprine effects on sarcoidosis has not been elucidated so far (73). Another second-line therapy option is mycophenolate mofetil (MMF), which inhibits the synthesis of purine nucleotides in lymphocytes and reduces the production of autoantibodies by B cells. MMF has been proven to be effective in small number of patients with pulmonary sarcoidosis, which manifested in pulmonary function improvement and nervous system effects. It has the lowest toxicity profile in all existing second-line therapy agents (74–76).

The third line includes the use of biological targeted therapy (77). Targeted TNF therapy was the first biological therapy used in patients with sarcoidosis. This is due to the fact that TNF-a is a product of macrophage activation that is a key pro-inflammatory cytokine that contributes to the spread of granulomatous inflammation. The role of TNF in disease progression has been demonstrated above. TNF-targeted therapy includes monoclonal antibodies (infliximab, adalimumab, golimumab), a recombinant protein that connects the TNF receptor to the permanent end of the IgG1 antibody (etanercept) and a pegylated Fab fragment of an anti-TNF-α humanized monoclonal antibody (certolizumab) (78). The drugs demonstrate various degree of effectiveness in different types of sarcoidosis; however, the available data are insufficient to develop a clear algorithm of their use. Infliximab is the most studied third-line drug. The recommended dose of infliximab together with maintenance therapy is 3–5 mg/kg administered every 4–8 weeks following the use of an initial loading dose. At the same time, the results of infliximab studies are somewhat contradictory and range from the evidence of improvement of lung lesions in 58.5% of patients to the lack of clinical significance (79, 80). The drug of choice for patients with infliximab resistance is adalimumab. Therapy with adalimumab was associated with an improvement during damage of skin and eye involvement, better pulmonary function, as well as reduced disease activity (81). At the same time, therapy with etanercept, a TNF receptor antagonist, has not been proven effective in sarcoidosis and therefore is not recommended. Neither golimumab [an anti-TNF-a human monoclonal antibody (IgG1)] nor ustekinumab (an IL-12 and IL-23 inhibitor) have been effective in respect of pulmonary function improvement, but have been shown to be partially effective in patients with sarcoid skin lesions (82).

Based on the hyper-expression of JAK-STAT pathway in granuloma cells the JAK-inhibitors were used in the treatment of patients with different types of sarcoidosis. The cases of efficient use of tofacitinib and ruxolitinib were described in pateints with cutaneous (47, 83), multi-organ (84), pulmonary sarcoidosis (85). The clinical studies of tofacitinib/ruxolitinib in sarcoidosis with/without corticosteroids (NCT03793439 and NCT03910543) are being conducted.

The described therapies can be beneficial for patients with weakened immune response, which can be diagnosed with the BAL or blood biomarkers, such as increased levels of neopterin, sIL-2R, chitotriosidase, neutrophil-derived collagenase and elastase (50), increase of cytokines (IL-13, CCL2, CCL18, TGF-β etc.) and a low level of TNF-α (51).

**Figure 3:**
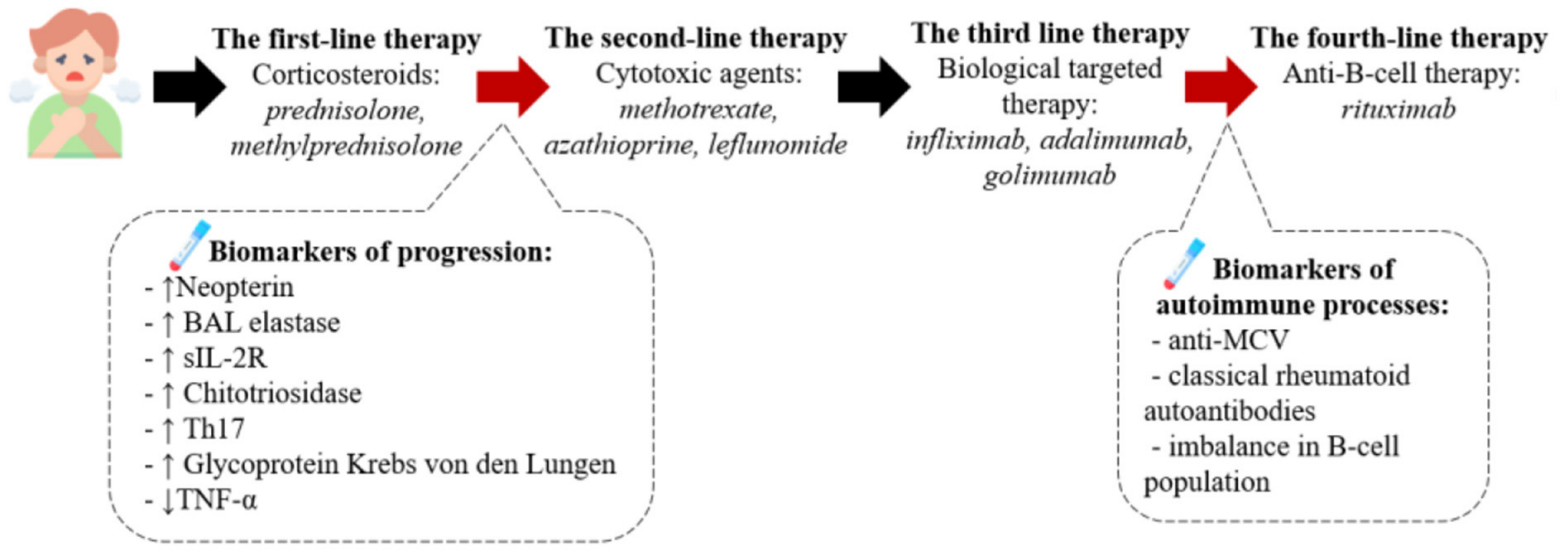
The pathogenesis-based tactic for sarcoidosis treatment. According to the clinical recommendations the first line therapy is corticosteroids, such as prednisolone or methylprednisolone. We assume the presence of biomarkers of the progression in blood or BALF can serve as criteria to start the second-line therapy presented by cytotoxic drugs (methotrexate, azathioprine etc.) and after – biological targeted therapy with anti-TNFα monoclonal antibodies. If the therapy has no beneficial effect we recommend to diagnose the presence of autoimmune processes (anti-MCV antibodies in the blood, the imbalance of B-cell population) and then consult with rheumatologist about the following treatment.

The described algorithm of the pathogenesis-based therapy of sarcoidosis is presented on Figure 3.

## Conclusion

Based on the results of this analysis of published data, the following characteristics of progressive chronic sarcoidosis were revealed: the disease develops in patients with genetic predisposition (SNP in genes GREM1, CARD15, TGF-β3, HLA-DQB1^*^06.02, HLA-DRB1^*^07/14/15) as a result of granulomatous inflammation following an antigen presentation in the lungs. It either causes immune system impairment or activates autoimmune disorders. Thus, the antigen elimination becomes impossible, and immune cells change their differentiation (Th1 to Th2, MP1 to MP2) during the chronic inflammation process and start producing cytokines (IL-13, CCL2, CCL18, TGF-β etc.) and enzymes (Arginase) resulting in fibroblast activation and the development of fibrosis.

**Figure 4:**
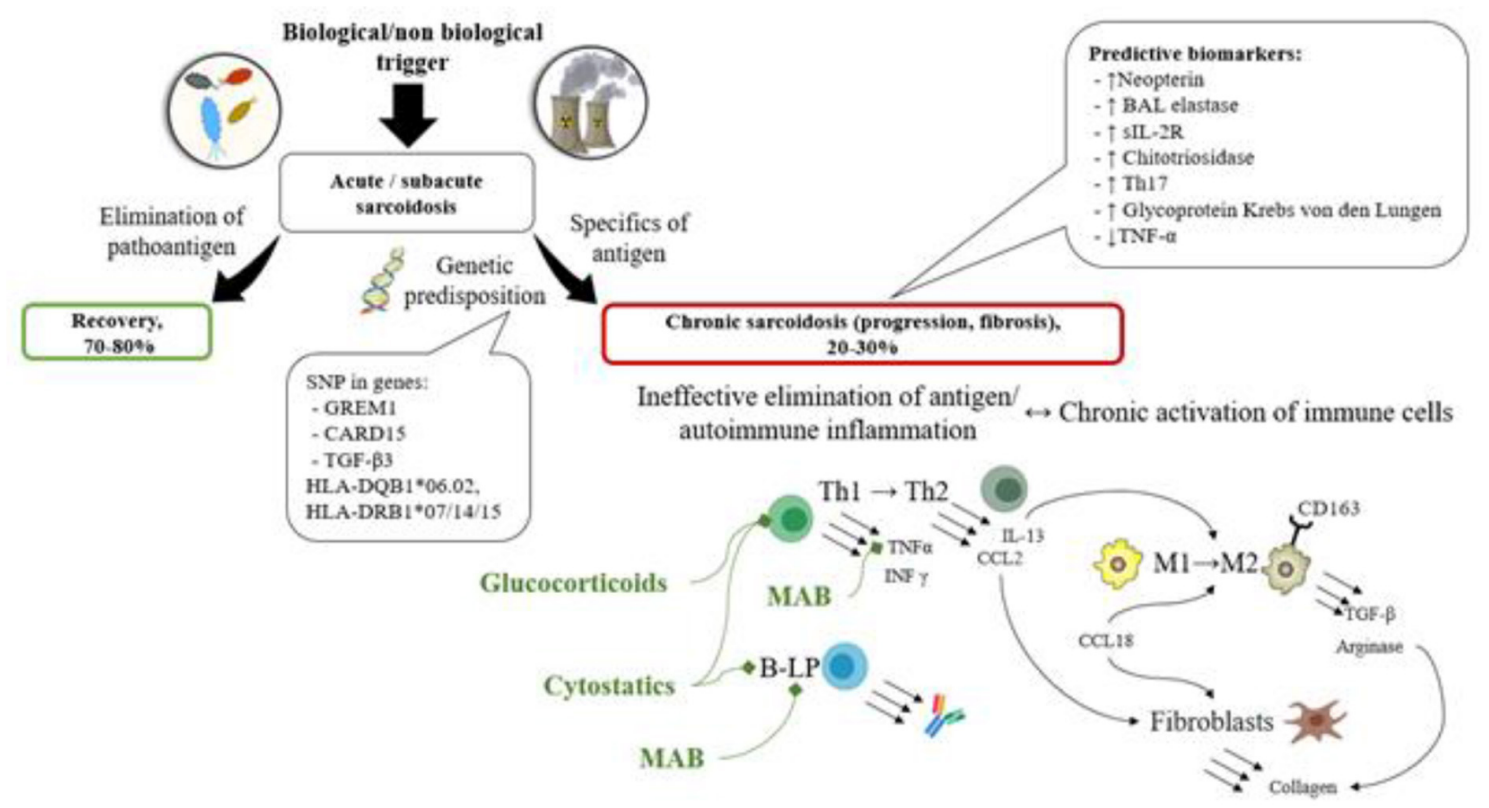
The scheme of the pathogenesis of sarcoidosis along with prognostic biomarkers, and treatment options based on the pathogenic processes. In patients with genetic predisposition (SNP in genes GREM1, CARD15, TGF-β3, HLA-DQB1*06.02, HLA-DRB1*07/14/15) immune system impairment is observed, characterized by immune response inefficacy or autoimmune potential. The antigen elimination becomes impossible, and immune cells change their differentiation (Th1 to Th2, MP1 to MP2) during the chronic inflammation process and start producing cytokines (IL-13, CCL2, CCL18, TGF-β etc.) and enzymes (arginase) resulting in fibroblast activation and the development of fibrosis. These processes can be represented by various prognostic biomarkers of disease progression (decreased levels of Neopterin, elastase, sIL-2R, Chitotriosidase, Glycoprotein Krebs von den Lungen, Th17 cell count, reduced quantity of TNF-α in peripheral blood or bronchoalveolar lavage fluid).

Various prognostic biomarkers of disease progression (increased levels of Neopterin, elastase, sIL-2R, Chitotriosidase, Glycoprotein Krebs von den Lungen, Th17 cell count, reduced quantity of TNF-α in peripheral blood or bronchoalveolar lavage fluid) have been described and can potentially be used to determine the group of patients who will benefit from the use of corticosteroids/ cytostatic drugs/biologics (see Figure 4).

However, currently it is difficult to determine which prognostic markers can be considered indicators for the use of corticosteroids/cytostatic agents/biologics, therefore clinical studies are needed to evaluate the diagnostic significance of determining the described genetic and laboratory markers for selecting a management strategy.

## Outstanding questions

At the moment, one of the problems of studying sarcoidosis is the lack of a clear connection of pathogenesis, a specific biomarker and the choice of treatment option. The solution of this practical issue can be aimed at: determining a group of patients with a low probability of self-limitation; pathogenetic justification for choosing a group of the 2nd and 3rd line therapy, based on the specific laboratory markers; determining patients at risk of relapse after drug withdrawal. For progress in these areas, it is necessary to continue research in the field of animal models of sarcoidosis, the connection of laboratory markers with variants of the course of the disease and their changes against the various therapy options, conducting clinical trials. This may make it possible to form clearer criteria for prescribing therapy, which in turn will increase its effectiveness.

## Author contributions

AM and IK conducted analysis of the materials and wrote the manuscript. AS conducted analysis of the materials, wrote the manuscript, and was a coordinator of the project. DK, YZ, and AG wrote the manuscript. PY was a coordinator the project and wrote the manuscript. YS was a coordinator of the project. All authors have read and agreed to the published version of the manuscript.

## Funding

This work was supported by the grant of the Government of the Russian Federation for the state support of scientific research carried out under the supervision of leading scientists, agreement 14.W03.31.0009.

## Conflict of interest

The authors declare that the research was conducted in the absence of any commercial or financial relationships that could be construed as a potential conflict of interest.

## Publisher's note

All claims expressed in this article are solely those of the authors and do not necessarily represent those of their affiliated organizations, or those of the publisher, the editors and the reviewers. Any product that may be evaluated in this article, or claim that may be made by its manufacturer, is not guaranteed or endorsed by the publisher.
